# Transmembrane protein CD9 is glioblastoma biomarker, relevant for maintenance of glioblastoma stem cells

**DOI:** 10.18632/oncotarget.5477

**Published:** 2015-11-11

**Authors:** Neža Podergajs, Helena Motaln, Uroš Rajčević, Urška Verbovšek, Marjan Koršič, Nina Obad, Heidi Espedal, Miloš Vittori, Christel Herold-Mende, Hrvoje Miletic, Rolf Bjerkvig, Tamara Lah Turnšek

**Affiliations:** ^1^ Department of Genetic Toxicology and Cancer Biology, National Institute of Biology, 1000 Ljubljana, Slovenia; ^2^ Department of Biochemistry, Blood Transfusion Centre of Slovenia, 1000 Ljubljana, Slovenia; ^3^ Department of Neurosurgery, University Medical Centre, University of Ljubljana, 1000 Ljubljana, Slovenia; ^4^ Department of Biomedicine, University of Bergen, 5009 Bergen, Norway; ^5^ Division of Neurosurgical Research, Department of Neurosurgery, University of Heidelberg, 69120 Heidelberg, Germany; ^6^ NorLux Neuro-Oncology Laboratory, Centre de Recherche Public de la Santé, 1526 Luxembourg, Luxembourg; ^7^ Department of Biochemistry, Faculty of Chemistry and Chemical Engineering, University of Ljubljana, 1000 Ljubljana, Slovenia

**Keywords:** biomarker, CD9, glioblastoma stem cells, neural stem cells, tetraspanin

## Abstract

The cancer stem cell model suggests that glioblastomas contain a subpopulation of stem-like tumor cells that reproduce themselves to sustain tumor growth. Targeting these cells thus represents a novel treatment strategy and therefore more specific markers that characterize glioblastoma stem cells need to be identified. In the present study, we performed transcriptomic analysis of glioblastoma tissues compared to normal brain tissues revealing sensible up-regulation of CD9 gene. *CD9* encodes the transmembrane protein tetraspanin which is involved in tumor cell invasion, apoptosis and resistance to chemotherapy. Using the public REMBRANDT database for brain tumors, we confirmed the prognostic value of CD9, whereby a more than two fold up-regulation correlates with shorter patient survival. We validated CD9 gene and protein expression showing selective up-regulation in glioblastoma stem cells isolated from primary biopsies and in primary organotypic glioblastoma spheroids as well as in U87-MG and U373 glioblastoma cell lines. In contrast, no or low CD9 gene expression was observed in normal human astrocytes, normal brain tissue and neural stem cells. *CD9* silencing in three CD133+ glioblastoma cell lines (NCH644, NCH421k and NCH660h) led to decreased cell proliferation, survival, invasion, and self-renewal ability, and altered expression of the stem-cell markers CD133, nestin and SOX2. Moreover, *CD9*-silenced glioblastoma stem cells showed altered activation patterns of the Akt, MapK and Stat3 signaling transducers. Orthotopic xenotransplantation of *CD9*-silenced glioblastoma stem cells into nude rats promoted prolonged survival. Therefore, CD9 should be further evaluated as a target for glioblastoma treatment.

## INTRODUCTION

Glioblastoma (GBM) is the most common primary brain tumor, as well as the most aggressive of primary gliomas. Despite modern treatments, following diagnosis, the overall median survival remains only about 15 months [1]. The hypothesis that a tumor contains tumor cells, acting as stem cells which are responsible for tumor development, was proposed more than a decade ago [2], and is still under further investigation [3–7]. GBM stem cells (GSCs) show characteristics of normal neural stem cells (NSCs), such as self-renewal and expression of the stem cell markers CD133, nestin and SOX2 [8, 9]. GSCs appear to show greater resistance to chemotherapy [10]. New approaches for GBM treatment are thus aimed at selective targeting of GSCs [11]. As most of the known stem cell markers are common to both GSCs and NSCs [12], novel selective GSC markers need to be identified to provide better targets for GBM therapies.

Since plasma membrane proteins are vital for the interaction of tumor cells with the micro-environment and downstream modulation of numerous biological processes, they account for almost two-thirds of the protein targets used in therapy of various pathological conditions [13]. Thus, along with the establishment of comprehensive omics data sets, a large number of studies have led to *in silico* searches of novel biomarkers that would also be therapeutic targets. Using various bioinformatic approaches, numerous up-regulated genes and proteins in GBM have been identified to represent potential theranostics, as they have been shown to be associated with tumor aggressiveness and shorter patient survival [14–16]. In this respect, the genes encoding transmembrane proteins are most suitable, due to their accessibility and ease of detection, as compared to intracellular proteins.

The tetraspanins represent a large family of plasma-membrane proteins. Tetraspanin CD9 is a 25-kDa transmembrane protein that has a role in cell invasion, apoptosis and resistance to chemotherapy, which are all key hallmarks of cancer [17]. There have been conflicting reports on CD9 expression, and it has been shown to be either increased [17, 18] or decreased, possibly acting as a tumor suppressor [19] in different cancer types including glioma [20]. Inverse correlation between CD9 expression and tumor cell invasion was shown for ovary cancer, cervical cancer and melanoma [17, 19]. When over-expressed, an increased migration and invasion of tumor cells were observed [21], as well as their reduced apoptosis induction, leading to increased resistance to chemotherapy [18, 22]. The mode of CD9 action depends on a number of its binding membrane associated proteins, increasing the variability of affected cellular functions. Thus, CD9 is known to form complexes with other tetraspanins, with receptor tyrosine kinases such as the epidermal growth factor receptor (EGFR) and the fibroblast growth factor receptor (FGFR), and with integrins (such as αvβ3 and others). Notably, CD9 can modulate their activities directly or via indirect binding to their ligands [17, 23, 24]. Binding of CD9 to receptor tyrosine kinases or their ligands has an important role in cell signaling. It was shown that a complex between CD9 and either HB-EGF or TGF-α, which are both EGFR ligands, leads to increased EGFR activation and consequently to increased activation of Ras/MapK and PI3K/Akt signaling pathways. Nonetheless, it was reported that a direct binding of CD9 to the extracellular domain of FGFR can also occur [23, 25, 26]. Different interactions between CD9 and other markers specific for oligodendrocyte precursor cells and the tumor niche components occur during development of different glioma subtypes. In a study revealing a model for identifying a cancer initiating cell, Liu and co-workers [27] reported a high expression of CD9 leading to the proneural subtype of glioma.

Here, we used a bioinformatics approach to search for genes that encode plasma membrane proteins, in particular cell surface receptors associated with kinase signaling, which are often overexpressed in GBM. The candidate gene CD9 met these criteria. In addition, our *in silico* investigations within the Repository for Molecular Brain Neoplasia Data (REMBRANDT) database, confirmed that *CD9* expression is increased in human GBMs, as compared to normal brain tissue. We confirmed the functional link with RTK signaling as some of the signaling transducers involved in EGFR and FGFR signaling pathways, i.e. MapK, Akt and Stat3 [28, 29] were affected by CD9 expression. In the same dataset, we also found that higher *CD9* expression correlates with shorter survival of GBM patients. Furthermore, we evaluated CD9 protein as a novel selective biomarker for GSCs, by determining its function, both *in vitro* and *in vivo*. We show that CD9 is involved in the regulation of survival and invasion of GSCs, as well as in stemness of GSCs, at least with respect to self-renewal and the expression of certain stem cell markers. We thus confirmed the contribution of CD9 to the malignancy of gliomas and GSCs, and propose to investigate CD9 further as a therapeutic target for GBM treatment.

## RESULTS

### Tetraspanin *CD9* mRNA was overexpressed in glioblastoma cells and glioblastoma stem cells compared to normal human astrocytes

The transcriptomic data deposited in the publicly available Data Portal of TCGA, GEO, and EMBL-EBI ArrayExpress were used to compare gene expression of NHAs with the U373 and U87-MG cell lines. These comparisons revealed 564 and 591 de-regulated genes for the U373 and U87-MG cells, with 262 and 246 genes to be up-regulated, respectively (Supplementary Table S1 and Supplementary Table S2). Among the de-regulated genes, the literature and the Biomine Search Engine were searched for any up-regulated genes that encode plasma membrane proteins, and that are putatively associated with receptor tyrosine kinases (RTKs), such as EGFR or FGFR, which are often overexpressed in GBM.

**Figure 1:**
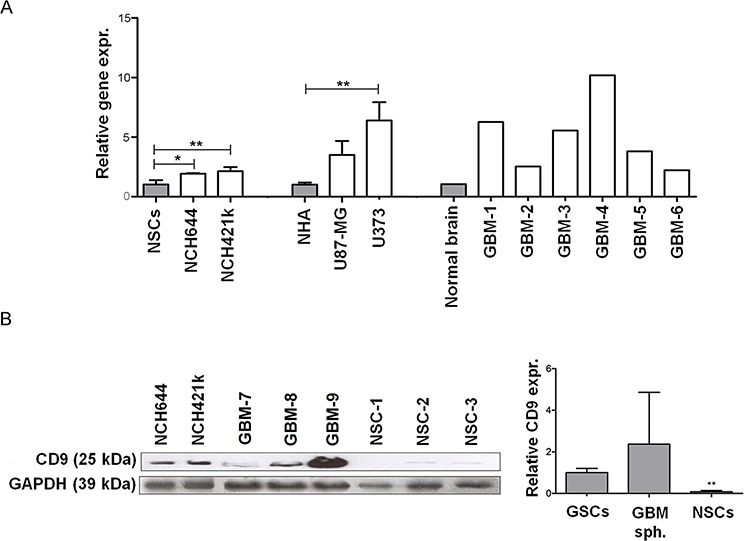
Expression of CD9 in GBM cells, GSCs cells and tissues **A.** *CD9* expression in two GSC lines (NCH644 and NCH421k) compared to NSCs as well as in the GBM cell lines (U373 and U87-MG) compared to NHAs and GBM tissues compared to normal brain tissue mix. Data represent means ±SD of three biological repeats (except for the 6 GBM tissue samples and commercial RNA from pooled normal brain tissue samples). **B.** Expression of CD9 protein in the GSC lines NCH644 and NCH421k, and three primary organotypic GBM spheroids (labeled GBM-7–9) enriched with GSCs and in three primary NSC cultures. GAPDH protein expression was used as a loading control. Representative images of three repeated experiments are shown on the left panel. The right panel shows the quantification of Western blotting for CD9 expression described above in two GSC lines NCH644 and NCH421k, three primary organotypic GBM spheroids and three primary NSC cultures, relative to the standard GAPDH protein. Quantification was performed using Image J software. Data are means ± SD of two (GSCs) and three (NSCs, GBM spheroids) samples; *: *p* < 0.05; **: *p* < 0.01.

CD9 gene satisfied these criteria, as it was up-regulated in U373 cells (although not in U87-MG cells), when compared to NHAs (Supplementary Table S4). CD9 protein was found to be a potential cancer stem cell marker in different types of cancer [30, 31]. Since CD9 is a novel protein and belongs to a family of tetraspanins which are involved in EGFR and FGFR signaling pathways [23, 32], we chose this protein for further validation and functional studies in GSC lines. Thus, CD9 was chosen mostly due to its potential biological functions in GBM stem cell biology where it has not yet been evaluated to a great extent. The *in silico* data for CD9 gene expression were validated by qPCR on cell cultures (Figure 1A). A 6.4-fold increase in *CD9* expression was confirmed in U373 cells compared to differentiated astrocyte line NHAs, whereas there was less, and no significant increase in U87-MG cells of CD9, perspectively. More consistent seems the *CD9* expression in both the NCH644 and NCH421k GSC lines, being 1.9-fold and 2.1-fold increased, respectively, when compared with nonmalignant counterpart, the less differentiated NSC line.

### Tetraspanin CD9 mRNA was up-regulated in glioblastoma tissue when compared to their non-malignant tissue counterparts

Transcriptomic data deposited in the publicly available TCGA Data Portal was used to compare gene expression of normal brain tissue *versus* GBM tissue. When compared to normal brain tissue, the GBM tissues showed 3521 genes that were de-regulated. Of these, 2118 were up-regulated, as given in Supplementary Table S3. As already indicated above, among the de-regulated genes, we searched for up-regulated plasma membrane genes that are associated with highly expressed the most abundant RTKs, the EGFR and/or FGFR genes in GBM. *CD9* was significantly up-regulated in GBM tissues, when compared to the normal brain (Supplementary Table S4). The qPCR validation of these *in silico* data on tissue cultures confirmed the average 5.1-fold increase in *CD9* expression in 6 samples of GBM tissue, compared to normal brain tissue mix (Figure 1A).

### Tetraspanin CD9 protein was overexpressed in glioblastoma cells and glioblastoma stem cells

CD9 protein expression was evaluated by Western blotting in two GSC lines (NCH644 and NCH421k), organotypic GBM spheroids, and primary NSC spheroids. The GSC lines showed high CD9 expression, whereas there was very low CD9 expression in the NSC spheroids. CD9 expression in 3 GBM spheroids was variable (Figure 1B, left panel). Western blot quantification showed approximately 10-fold and about 20-fold higher CD9 expression in GSCs compared to 3 NSCs and 3 GBM samples (Figure 1B, right panel). Taken together, these data demonstrate CD9 overexpression at the mRNA and protein levels in some GBM cell lines and in all GSCs used in this study, and very low CD9 gene and protein expression in NHAs and NSCs.

### *CD9* silencing decreased stemness of glioblastoma stem cells

#### Lentiviral shRNA silencing of CD9

To analyze the function of CD9 in GSCs, its expression was down-regulated by *CD9*-directed lentiviral shRNA transduction in three GSC lines, NCH644, NCH421k and NCH660h cells. These *CD9*-silenced cells were compared to the control non–*CD9*-silenced cells, which were transduced with a scrambled shRNA. Although *CD9* shRNA viral transduction was not 100% efficient as confirmed by qPCR and Western blotting, it led to a significant reduction of CD9 gene and protein expression as compared to the controls (Supplementary Figure S1), and thus the effect of *CD9* down-regulation was functionally evaluated. Incomplete *CD9* silencing might be a result of the fact that a few DNA copies of the viral genome were integrated into the chromosomal regions for antibiotic resistance. As a consequence, integration of these copies did not result in *CD9* silencing but only in resistance to the antibiotic. Consequently, the transduced cells survived in the selection medium with the antibiotic although no *CD9* silencing occurred.

#### Stem cell markers gene expression

**Figure 2:**
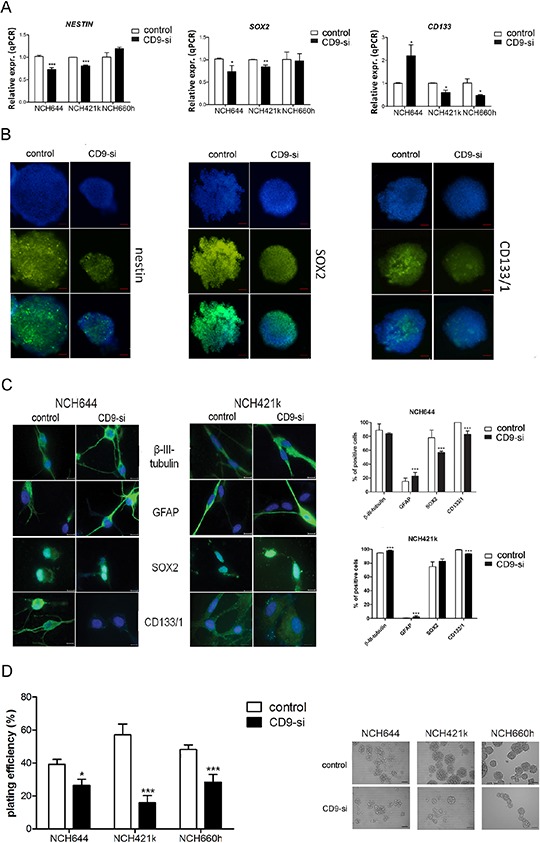
*CD9* silencing effects on stem cell marker expression The effects of *CD9* silencing on stem cell markers were analyzed in three *CD9*-silenced GSC lines (“CD9-si”), as compared to the non-*CD9*-silenced cells (“control”). **A.** Gene expression of stem cell markers nestin, SOX2 and CD133 was measured by qPCR in three GSC lines NCH644, NCH421k and NCH660h. **B.** Nestin, SOX2 and CD133/1 protein expression analysis in the *CD9*-silenced NCH644 cell line. Blue, cell nuclei; green, proteins, as indicated. Representative images of three repeated experiments are shown for both channels (blue, green) as well as for overlays. Scale bar, 100 μm. **C.** Immunocytochemistry detection in the *CD9* silenced GSC lines NCH644 and NCH421k was performed for detection of differentiation protein markers, such as glial fibrillary acidic protein (GFAP) and b-III-tubulin, related to glial and neuronal differentiation, respectively as well as for detection of stem cell markers, such as CD133/1 and SOX2. Representative images of two repeated experiments are shown. Scale bar, 100 μm. Quantification of the results is shown on the right panel. Two visible fields of each staining were evaluated at 200 × magnification. Data are means ±SD of two independent experiments. **D.** Clonogenic assay for self-renewal of the *CD9*-silenced GSC lines. Data are means ± SD of three independent experiments. Representative data of each cell line are also shown. Scale bar, 100 μm. *, *p* < 0.05; **, *p* < 0.01; ***, *p* < 0.001.

The influence of *CD9* silencing on the expression of the stem cell markers nestin, SOX2 and CD133 was investigated using qPCR (Figure 2A). Compared to the control non–*CD9*-silenced cells, nestin expression decreased by 29% and 19% in *CD9*-silenced NCH644 and NCH421k cells, respectively, and *SOX2* expression was also decreased by 28% and 16% in these cells, respectively. For *CD133* expression, this was increased in the *CD9*-silenced NCH644 cells, but decreased by 39% in the *CD9*-silenced NCH421k cells and by 52% in the *CD9*-silenced NCH660h cells.

#### Stem cell marker protein expression

Nestin, SOX2 and CD133/1 expression was also investigated in these GSCs on a protein level, by immunocytochemistry, which showed less CD133/1 protein in all three *CD9*-silenced GSC lines, compared to the expressions were control non–*CD9*-silenced GSCs (Figure 2B for NCH644 cell line; Supplementary Figure S2 for NCH421k and NCH660h lines). Similarly, nestin and SOX2 protein expression were decreased upon *CD9* silencing, thus correlating with their gene expression levels in all three *CD9*-silenced GSC lines.

#### Differentiation assay

Immunocytochemistry detection in the *CD9* silenced GSC lines NCH644 and NCH421k was performed for detection of the differentiation protein markers, possibly induced upon *CD9* down-regulation, such as glial fibrillary acidic protein (GFAP) and b-III-tubulin which are related to glial and neuronal differentiation, respectively (Figure 2C). GFAP expression increased in both differentiated *CD9*-silenced GSC lines, whereas b-III-tubulin increased only in differentiated *CD9*-silenced NCH421k cells as compared to the control cells. As expected, expression of CD133/1 decreased in both cell lines, whereas SOX2 expression decreased only in the differentiated *CD9*-silenced NCH644 cell line.

#### Clonogenic assay

An important hallmark of cancer stem cells is their self-renewal capacity. In the present study, we performed a clonogenic assay to determine whether *CD9*-silenced GSCs can form spheroids from a single cell embedded in an agarose matrix. Upon *CD9* silencing, spheroid formation was decreased by 33%, 72% and 41% in NCH644, NCH421k and NCH660h cells, respectively, as compared to the control non–*CD9*-silenced cells (Figure 2D). These data imply that CD9 is involved in the maintenance of certain GSC features like stem cell marker expression and self-renewal.

### *CD9* silencing in glioblastoma stem cells led to reduction in key cell processes associated with glioblastoma malignancy

#### Cell proliferation

**Figure 3:**
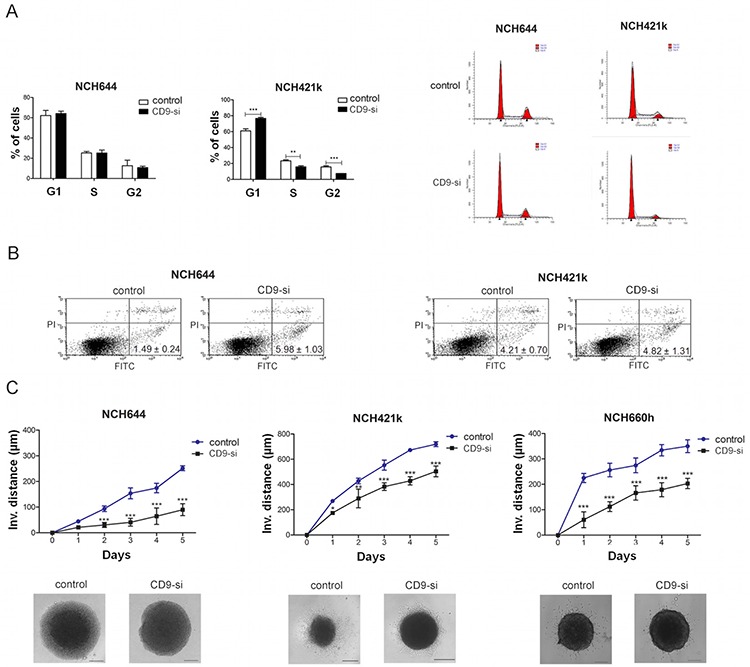
*CD9* silencing decreased survival and impaired invasion of GSCs The effects of *CD9* silencing on proliferation, apoptosis and invasion of the GSC lines (“CD9-si”) was measured and compared to the non *CD9*-silenced cells (“control”). **A.** Cell cycle analysis showing percentage of cells in each phase of the cell cycle (left). A representative histogram of each cell line (control and *CD9*-silenced) is shown on the right. **B.** FACS analysis for Annexin V FITC and propidium iodide staining to evaluate *CD9* involvement in apoptosis. Percentages represent the early apoptotic cells (Annexin V FITC labeled; upper left quadrant). Necrotic cells (PI labeled) are located in the lower right quadrant and late apoptotic cells (Annexin V FITC and PI labeled) are located in the upper right quadrant. Data are means ±SD of three independent experiments. **C.** Invasion of *CD9*-silenced GSCs from spheroids embedded in a collagen matrix as measured by invasion distance of out-going cells determined up to 5 days under a microscope. The invasion distance was defined as the distance from the edge of the spheroid to the most distant cell population. A representative of each cell line (control and *CD9*-silenced) is also shown. Scale bar, 200 μm. Data are means ±SD of three independent experiments. *, *p* < 0.05; **, *p* < 0.01; ***, *p* < 0.001.

The effect of CD9 on GSC proliferation was determined by cell cycle analysis (Figure 3A). *CD9* silencing in NCH421k cells significantly increased the number of cells in G1 phase by 1.3-fold and decreased the number of cells in S phase and G2M phase by 1.5-fold and 2.1-fold, respectively, indicating that CD9 may promote the transition from G1 to S and cell proliferation. Yet, in NCH644 cells no significant effects of *CD9* silencing on the cell cycle was noted. For confirmation of the proliferation results, MTS viability assay was performed which showed a decrease in percentage of viable *CD9*-silenced NCH421 cells by 26%, when compared to the control non-*CD9*-silenced cells (Supplementary Figure S3).

#### Cell survival

For the evaluation of CD9 involvement in cell death, we performed Annexin V FITC and propidium iodide staining of the *CD9*-silenced and non–*CD9*-silenced GSC lines (Figure 3B). The percentages of the *CD9*-silenced NCH644 cells that were stained only with Annexin V (early apoptotic cells) was 4.0-fold higher compared to the control non–*CD9*-silenced NCH644 cells, whereas no effects were noted for the *CD9*-silenced NCH421k cells. Taken together, these data suggest that CD9 in NCH421k regulates proliferation of these cells, whereas in NCH644 it impairs the apoptosis; both processes being relevant for stabilizing the GSCs in a characteristic low proliferating and apoptosis resistant state, although these regulations seem to be to some extent dependent on the biological origin of GSCs, as observed differences do exist in both types of NCH cell lines.

#### Cell invasion

The impact of *CD9* silencing on the invasion of GSCs was investigated using a three-dimensional collagen-embedding assay. The distances of the invading cells from a spheroid were measured on five consecutive days (Figure 3C). *CD9* silencing decreased invasion of NCH644, NCH421k and NCH660h cells by 35%, 67% and 45%, respectively, when compared to the non–*CD9*-silenced cells. These data indicate that CD9 enhances the invasive properties of the GSCs.

### *CD9* silencing changed intracellular kinase signaling

**Figure 4:**
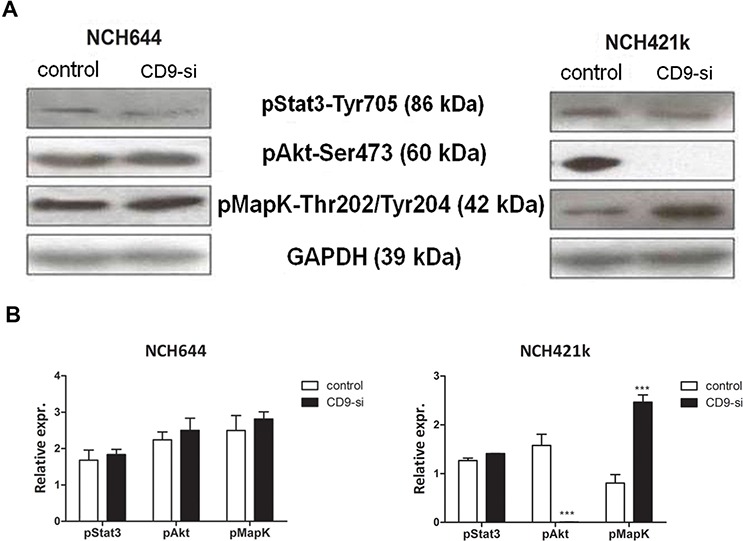
*CD9* silencing affects the expression of signaling transducers in GSCs The effects of *CD9* silencing on receptor tyrosine kinase signaling in GSC lines (“CD9-si”) was measured and compared to the non-*CD9*-silenced cells (“control”). **A.** Representative Western blotting for expression of three phosphorylated signaling tranducers Akt, Map kinase and Stat3 in two GSC lines upon *CD9* silencing. GAPDH is included as an internal control. **B.** Quantification of Western blotting for relative expression of signaling transducers in two GSC lines upon *CD9* silencing. Data are means ± SD of three independent experiments. ***, *p* < 0.001.

Tetraspanin association with other plasma-membrane proteins might lead to an activation of some of the key signaling transducers. In terms of phosphorylation of Akt, MapK and Stat3, protein levels were thus analyzed in two *CD9*-silenced GSC lines using Western blotting (Figure 4). Akt kinase activity was completely abolished and an approximately 2-fold up regulation of Map kinase was noted in the *CD9*-silenced NCH421k cell line. In contrast, *CD9* silencing in NCH644 cells had no significant effects on growth factor receptors levels and Akt, MapK or Stat3 signaling pathways. Taken together, these data imply the existence of trans-membrane CD9 interactions with the signaling pathways' proteins, where expression and activation of these signaling transducers is affected in different ways in both NCH cell lines.

### *CD9* had an impact on cell survival

#### CD9 in prognosis of survival of glioblastoma patients

**Figure 5:**
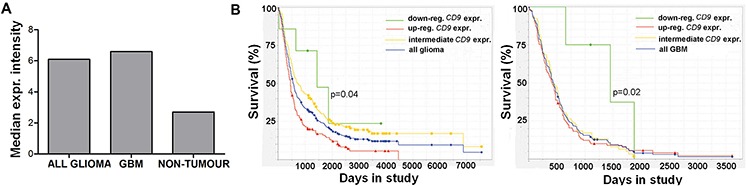
*CD9* expression affects prognosis of glioma patients **A.** Analysis of the REMBRANDT database query for *CD9* expression in 198 glioma and 228 GBM samples, and 28 non-tumor samples. **B.** The REMBRANDT database was also analyzed for the relationship between *CD9* expression and median survival of the patients with glioma and GBM. Kaplan–Meier curves for 7 glioma patients with > 2.0 fold down-regulation of *CD9* expression compared to the > 2.0 fold up-regulated group of 161 glioma patients (left panel) and for 4 GBM patients with > 2.0 fold down-regulation of *CD9* expression compared to 76 GBM patients in the intermediate group (right panel).

Gene expression analysis using the REMBRANDT database tool revealed increased *CD9* expression in gliomas, including GBM, compared to normal brain (Figure 5A). The survival queries revealed that for the 161 patients with glioma that showed > 2.0-fold up-regulation of *CD9*, there was a 50% shorter median survival, compared to the group of seven glioma patients with 2.0-fold down-regulation of *CD9* (Figure 5B, left; 24 months vs. 48 months, respectively; *p* = 0.04). Furthermore, the 76 patients with GBM that showed > 2.0-fold up-regulation of *CD9* showed a 55% shorter median survival compared to the intermediate patient group, as those with CD9 gene expression between 2.0-fold up-regulated and 2.0-fold down-regulated (Figure 5B, right; 19 months vs. 42 months, respectively; *p* = 0.02). These data indicate that *CD9* up-regulation in GBM patients has an important contributed to their shorter survival.

#### CD9 silencing inhibited tumor growth *in vivo*

**Figure 6:**
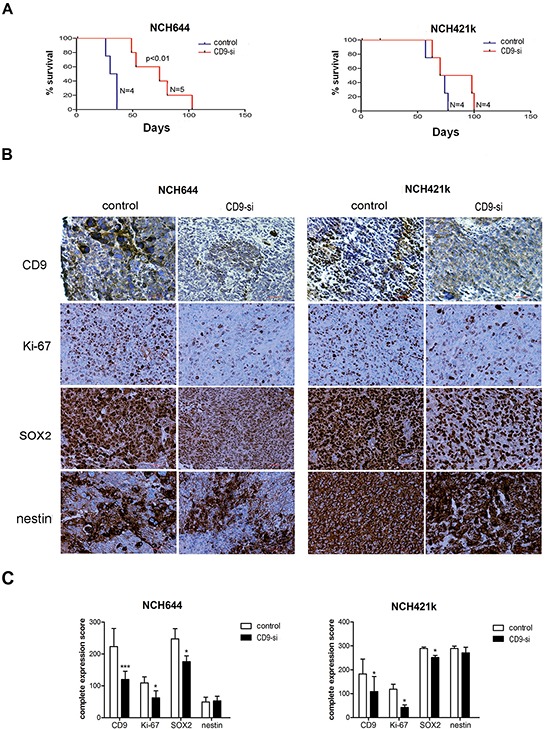
*CD9* silencing prolongs survival of rats and alters protein expression of selected biomarkers in xenografts Spheroids from *CD9*-silenced NCH644 and NCH421k (“CD9-si”) and the non-*CD9*-silenced cells (“control”) were implanted into the brains of nude rats to determine the effects of *CD9* silencing on survival. **A.** Kaplan–Meier curves for *CD9*-silenced GSC lines compared to the non-*CD9*-silenced GSC lines. **B.** Representative immunohistochemistry staining for expression of CD9, proliferation marker Ki-67, stem cell markers nestin and SOX2. Scale bar, 200 μm. **C.** Quantification of immunohistochemistry staining of paraffin sections from *CD9*-silenced and non-*CD9*-silened xenografts. Complete expression scores were calculated as mean percentage of positive cells throughout the whole tumor area multiplied by the mean staining intensity (0, negative; 1, weak; 2, moderate; 3, strong). *, *p* < 0.05; **, *p* < 0.01; ***, *p* < 0.001.

Spheroids of two *CD9*-silenced and non–*CD9*-silenced GSC lines (NCH644, NCH421k) were orthotopically implanted into nude rats. The rats from the *CD9*-silenced NCH644 cell group showed a 2.2-fold longer median survival (Figure 6A; 74 days vs. 33 days, respectively; *p* = 0.004). Although not significant, the survival of rats inoculated with the spheroids of the *CD9*-silenced NCH421k cells was 1.2-fold longer as compared to the control group (Figure 6A; 84 days vs. 72 days, respectively; *p* = 0.289).

Immunohistochemistry of xenografts confirmed decreased expression of *CD9* in both *CD9*-silenced NCH644 and NCH421k cell xenografts, together with significantly decreased expression of the proliferation marker Ki-67 (by 43% and 65%, respectively) as shown in Figure 6B, 6C. This is in line with the results of the cell cycle arrest and higher percentage of early apoptotic NCH421k and NCH644 cells, respectively, as found *in vitro*. Similarly to *in vitro*, the stem cell marker SOX2 protein was decreased in *CD9*-silenced tumors (by 29% and 13%, respectively), whereas nestin was not.

## DISCUSSION

New promising approaches in GBM treatment need to consider targeting of GSCs [11], and thus the aim of the present study was to identify and determine the function of a putative novel GSC target protein in GBM. In the search for such proteins that are not expressed by NSCs as well [33], we focused on plasma membrane-associated gene products, as these might have better therapeutic potential in a clinical context. This is the first study on reporting of differential expression of plasma membrane biomarker CD9 in GBM stem cells vs. normal neural stem cells (NSC), poly present in the GBM tissues, and thus representing novel possibilities of selective biological targeting. Our transcriptomic analyses pointed to CD9 gene, which codes for the tetraspanin cell surface protein, thus being of high relevance and of interest here as a novel GBM biomarker. We found up-regulated CD9 gene in GBM tissues and U373 cells, in comparison with normal brain tissue and NHAs, respectively. This is in line with the increased levels of CD9 transcript in GBM tissue reported by Kawashima et al. [34], who also confirmed a correlation between CD9 expression and astrocyte tumor malignancy in a cohort of 96 patients. CD9 protein was found to be a potential cancer stem cell marker in different types of cancer [30, 31]. Since it belongs to the family of tetraspanins that are involved in RTK signaling, highly relevant not only for GBM but also for GSCs which are known to retain their stem cell characteristics in the EGF and FGF-2 enriched Neurobasal medium [23, 32], we chose this protein for further validation and evaluation. Thus, CD9 was chosen mostly due to its potential biological functions in GBM stem cell biology where it has not yet been evaluated to a great extent.

In this study, we used GSC lines, NCH644 and NCH421k [35, 36] derived from primary GBMs, belonging to proneural category [37] and showing typical GBM aberrations, as indicated previously by DNA copy-number profiling by array-CGH [37]. NCH421k additionally carried amplifications of the PDGFRA and CDK4 gene loci but both primary tumors lack amplification of the EGFR gene locus [35, 36]. Since the results obtained with the two cell lines differed in some cases, we used an additional GSC line NCH660h to confirm the results of the most critical experiments. *CD9* expression was validated here by qPCR in a series of these heterogeneous, although well characterized GSC lines and we found significantly higher *CD9* expression in them *c*ompared to the normal fetal neural stem cells (NSCs), on both the mRNA and protein levels. There has been a similar report from Engström et al. [14], who identified *CD9* as one of the top five candidates to distinguish GSCs from NSCs in high-throughput sequencing of transcript tags (Tag-seq) performed on adherent GBM-derived NSCs and normal NSCs. The association of CD9 expression with stem-cell characteristics of GSCs was also reported by Lottaz et al. [37], who demonstrated significantly lower *CD9* expression in fetal NSCs compared to CD133^+^ GSC lines. Gene expression changes underlying glioma development have already enabled classification of GBM into four distinct molecular types, thus indicating the need for different treatment regimens, including potential targeting of these GSCs of different origins [14]. The transcriptomic analysis by Lottaz et al. [37] clearly distinguished between two types of GSCs. Type I GSCs have a ‘proneural’ gene signature that resembled fetal NSCs, whereas type II GSCs showed a ‘mesenchymal’ transcription profile that was similar to adult NSCs. Phenotypically, type I GSCs are CD133^+^ positive and grow as neurospheres, and are thus similar to the CD133^+^ NCH644, NCH421k and NCH660h GSC lines used in the present study. However, we observed variability in CD9 expression among these lines, coinciding with their differences in proliferation and their apoptotic tendencies [32]. These can be ascribed to their plasticity [38], reportedly existing even within the same tumor [39]. Variable CD9 expression in the primary GBM spheroids grown under stem cell enriching conditions, as observed in the present study, might otherwise be due to different inherent abundance of GSCs in the original tumors [40, 41]. Moreover, the previously noted variability in the gene expression profiles of established GBM cell lines [42] is also reflected in the present study where *CD9* expression was significantly increased in the U373 cells, but was not changed in the U87-MG cells, compared to normal astrocytes.

The functional validation of CD9 using *CD9*-directed shRNA silencing resulted in changed GSC phenotypes of the NCH644, NCH421k and NCH660h cell lines. With respect to stemness, *CD9* silencing significantly affected the mRNA expression of the most relevant stem cells markers CD133, nestin and SOX2 and this was associated with loss of GSC self-renewal. Decreased stem cell markers' expression upon *CD9* silencing is not necessarily a direct effect of a decreased CD9 expression. These responses might be a direct, causative, or an indirect associated effect (late gene response) to silencing or might even be an off-target effect. Nevertheless, our findings support but does not prove the concept that CD9 represents a GSC biomarker. Also, in rat xenografts derived from *CD9*-silenced NCH644 and NCH421k cells, there was significantly decreased expression of SOX2, but not of nestin, which suggests the involvement of CD9 in the maintenance of cell stemness during de-differentiation to a malignant GBM. Also, in other tumors, CD9 has been reported as a cancer stem cell biomarker, for example in mesothelioma and in lymphoblastic leukemia cells [30, 31].

With respect to other characteristics of the stem cell phenotype, we are showing here that CD9 can mediate proliferation, survival and invasion of GSCs *in vitro*. Although in all tested GSC lines *CD9* silencing impairs invasion, the cells differed in the role that CD9 may have in proliferation and survival by either promoting the G1- S phase transition in NCH412k or inhibiting the NCH4644 cells apoptosis, respectively. Since Ki-67 staining of IHC xenografts clearly showed decreased proliferation of both *CD9*-silenced NCH cell lines, it is possible that increased percentage of *CD9*-silenced NCH644 cells in G1-S transition might be due to an increase or redistribution of the G0 fraction and reduction of cells entering the G1 phase.

Based on our signaling cascade alteration upon *CD9* silencing, one possible explanation may be that by inhibiting Akt activation in the NCH421k line, CD9 decreased its proliferation and invasion. However, the signaling pathways alterations in NCH644 were different, possibly acting *via* other kinase affecting apoptosis. This appears to be through mechanisms that involve its rather promiscuous binding and activation of several CD9 protein membrane partners, such as integrins [43] and RTKs or their ligands, to allow for different signaling pathways *via* those signaling transducers [44]. However, the observed *CD9* silencing efficiency variations among the cell lines is less likely reflected in such as drastic effect on the phosphorylation activities of the kinases. We rather hypothesize that these differences are due to the fact that these GSC lines originate from genetically different GBM subtypes, also exhibiting different signaling pathways' activation. Noteworthy, NCH421k and NCH644 indeed differ in PDGFRα expression which may also play yet unknown role in the CD9 associated signaling [37].

The correlation analysis of the survival and gene expression data of patients with GBM performed here within the REMBRANDT database revealed that patients with higher *CD9* expression had shorter survival. As we observed strong correlation between *CD9* expression and expression of known stem-cell markers in the *CD9*-silenced GSCs, the worse prognosis for patients with GBM that shows higher *CD9* expression might be explained by GBM resistance to therapy, possibly due to a higher fraction of GSCs [10]. Similarly, CD133, another putative stem cell marker, was confirmed as prognostic for shorter overall survival of patients with GBM [45–47]. In addition, Strojnik et al. [46] demonstrated that nestin has a significant impact on survival for patients with GBM, whereas Annovazi et al. [48] confirmed a correlation between stem cell marker *SOX2* expression and glioma malignancy grade. This is in line with the xenografts derived from spheroids of the two *CD9*-silenced GSC lines that were orthotopically implanted into nude rats in the present study, where we found a strong correlation between *CD9* and *SOX2* expression. Kawashima et al. [34] transplanted glioma cells into *CD9*-deficient mice and observed that tumor-associated CD9 expression is relevant for malignancy of tumor cells independently of their micro-environment, thus supporting our data. The authors suggested that this was due to CD9-mediated acceleration of mitogenic and adhesive properties of GBM cells. In our animal study, the down-regulation of *CD9* in both GSC lines also prolonged the survival of a small cohort of animals; although significant only for the NCH644 cell line, but implying on a prognostic potential of CD9. Since the silencing of *CD9* in the NCH421k cell line was less effective in comparison with the NCH644 cell line, this could also affect the survival of rats in our experiment. As invasion correlated with CD9 levels in all GSC lines used in this study, we would rather suggest that inhibition of invasion had stronger impact on survival of patients and rats, as the effects on proliferation and apoptosis were not consistent.

In summary, we have shown here the relevance of CD9 as a prognostic GSC marker that can allow discrimination between GSCs and NSCs. Our *in vitro* and *in vivo* study demonstrate that CD9 is relevant for maintenance of their stemness potential and for invasion, although its role in GSC proliferation and survival is variable and possibly depends on the biological and genetic background of the cells. The *CD9* up-regulation may enhance resistance of GBM to standard therapy as we found it prognostic and predictive for GBM patients' survival. However, further *in vivo* studies on CD9 targeting are needed to confirm its applicability in the treatment of GBM.

## MATERIALS AND METHODS

### Cell lines and tissue culture

#### Cell lines

The U373 and U87-MG human GBM cell lines were from the American Type Culture Collection and were cultured in high-glucose (4.5 g/L D-glucose) Dulbecco's modified Eagle's medium (DMEM; Invitrogen, Life Technologies), supplemented with 10% fetal bovine serum (FBS), 2 mM L-glutamine and 1 × penicillin/streptomycin (all from PAA Laboratories). Authentication of these cell lines was performed by DNA fingerprinting, using Amp-FlSTR Profiler Plus PCR Amplification Kit, as described previously [49, 50]. Normal human astrocytes (NHAs) were from Lonza, and were cultured in low-glucose (1.0 g/L D-glucose) DMEM (Sigma-Aldrich) supplemented with 10% FBS, 2 mM L-glutamine, 1 × penicillin/streptomycin, and 20 nM HEPES (Gibco, Life Technologies). Three previously established CD133^+^ GSC lines, NCH644, NCH421k and NCH660h [35, 36], were grown as spheroid suspensions in complete Neurobasal Medium (Invitrogen, Life Technologies) containing 2 mM L-glutamine, 1 × penicillin/streptomycin, 1 × B-27 (Invitrogen, Life Technologies), 1 U/mL heparin (Sigma-Aldrich), 20 ng/mL bFGF and EGF (both from Invitrogen, Life Technologies). Once these GSC spheroids reached 200 μm in diameter, they were dissociated mechanically. These cell lines were authenticated at the Leibniz Institute DMSZ (Braunschweig, Germany) by direct comparison with the original patient tissue (short tandem repeat profiles available upon request), and they were used within 6 months of authentication.

#### Primary neural stem cell cultures

Fetal, non-malignant, post-mortem brain tissue was obtained from the University Medical Centre, University of Ljubljana, according to Ethical Committee Approval Number 156/07/09, and used for the establishment of primary NSC cultures. The brain tissue was cut into small pieces, treated with trypsin/EDTA (Invitrogen, Life Technologies), and filtered through 40-μm nylon filters (BD Biosciences). The cells were then grown in complete Neurobasal Medium, as described above. These NSCs were used for further experiments after two weeks in culture.

#### GBM tissue samples

Brain tissue samples from patients with GBM were obtained from the Department of Neurosurgery of the University Medical Centre, University of Ljubljana, according to Ethical Committee Approval Number 109, 204–6/10/07. These tumor samples were either used for the generation of organotypic cultures or were frozen upon tumor removal for RNA extraction.

#### Organotypic GBM tissue cultures

Primary spheroid cultures generated from the human GBM tissue samples were cut into small fragments and cultured on agar-coated plates in DMEM (4.5 g/L D-glucose) supplemented with 10% FBS, 1 × L-glutamine, 1 × penicillin/streptomycin and 25 × non-essential amino acids (Sigma-Aldrich). The resulting organotypic GBM spheroids were used for further experiments after two weeks in culture.

### Gene expression analyses of cell lines

Trancriptomic analysis was carried out for the NHA cell line which was compared to those for the two GBM cell lines, U373 and U87-MG, as described by Verbovšek et al. [51]. Briefly, raw gene-expression data for the NHAs and these GBM cell lines were downloaded from the publicly available website of the *Gene Expression Omnibus* (GEO), European Molecular Biology Laboratories (EMBL) – European Bioinformatics Institute (EBI) ArrayExpress (http://www.ebi.ac.uk/arrayexpress/), as: GSE12305 (samples GSM309429, GSM309431, GSM309432) and GSE9834 (samples GSM247617, GSM247618, GSM247619) for the NHAs (downloaded on June 21^st^, 2011); E-MEXP-903 (samples U373D-1, U373D-2, U373D-4) for the U373 cells (downloaded on November 17^th^, 2011); and GSE18494 (samples GSM460681, GSM460682, GSM460683) for the U87-MG cells (downloaded on July 16^th^, 2010). Data for GBM and normal brain tissue (24 samples for GBM tissue and 10 samples for unmatched control brain tissue) was obtained in July 2011 from The Cancer Genome Atlas (TCGA) Data Portal (https://tcga-data.nci.nih.gov/tcga/). As these data were merged from multiple studies, and thus different experimental sources, we used RankProd [52], a Bioconductor package for detection of differentially expressed genes. De-regulated genes with a percentage of false predictions < 0.05 were identified for the U373 and U87-MG cells, for comparison with those for the NHAs.

### Gene expression analyses of glioblastoma and normal tissue

Transcriptomic analysis was also performed for the GBM tissue, in comparison with normal brain tissue, as described by Verbovšek et al. [51]. Briefly, raw gene expression data for normal brain tissue and GBM tissue were collected from the Cancer Genome Atlas (TCGA) Data Portal (https://tcga-data.nci.nih.gov/tcga/), as Batch 8: 24 tumor samples and 10 unmatched normal brain samples. As these data were merged from different experimental sources, the RankProd [52] Bioconductor package was used. As compared to normal brain tissue, the de-regulated genes in the GBM tissue were identified, for a percentage of false predictions < 0.05.

### Survival analyses of patients with glioma

*CD9* gene expression analysis of the glioma tissue and its association with the survival of these patients with GBM was performed using the REMBRANDT public database for brain tumors [53]. The queries were carried out with the REMBRANDT ‘simple search’ option, using 454 samples (the 201005 probe set) where the *CD9* expression query included 228 patients with GBM, 198 patients with other grades of glioma, and 28 non-tumor patients. Before the survival queries, the patients were categorized based on their *CD9* expression as 2-fold down-regulated (D) or 2-fold up-regulated (U), with the values between these restrictions defined as intermediates. The first survival query was performed with all glioma patients (*N* = 343; *U* = 161, *D* = 7), and the second survival query was performed with the patients with GBM (*N* = 181; *U* = 101, *D* = 4).

### mRNA expression analysis

The cultured tissues and cell lines were washed with PBS (PAA Laboratories) and their total RNA was isolated using TRIzol reagent (Invitrogen, Life Technologies), according to the manufacturer instructions. In addition, a commercially available normal brain tissue pool of mRNA was used (FirstChoice Human Brain Reference RNA; 23 pooled samples; Applied Biosystems, Life Technologies). Reverse transcription of 500 ng total RNA was performed using cDNA High Capacity Archive Kit (Applied Biosystems, Life Technologies), following the manufacturer protocol. Gene expression was quantified using real-time quantitative PCR (qPCR; ABI 7900 HT Sequence Detection System, Applied Biosystems). TaqMan Universal PCR Master Mix and the following Taqman Gene Expression Assays were used: *CD9* (CD9 molecule, Hs00233521_m1), *PROM1* (prominin 1/CD133, Hs00195682_m1); *NES* (nestin, Hs00707120_s1), *SOX-2* (sex determining region Y-box 2, Hs01053049_s1 and *GAPDH* (glyceraldehyde-3-phosphate dehydrogenase, Hs01076091_m1) as an internal control (all from Applied Biosystems, Life Technologies).

The conditions for the qPCR were 50°C (2 min), 95°C (10 min) and 40 cycles of 95°C (15 s) and 60°C (1 min). The data obtained were analyzed according to the ΔΔCt algorithm, and are expressed as relative gene expression. The experiments were performed as three biological repeats, except for six GBM tissue samples and the commercial pool of RNA from the normal brain tissue samples.

### Western blotting

The GSC spheroids and organotypic GBM spheroids were washed with PBS and lysed in buffer (50 mM Tris, pH 6.9, 0.05% Brij 35, 0.5 mM dithioerythritol, 5 mM EDTA, 0.5 mM phenylmethylsulphonyl fluoride and 10 μM pepstatin A). Protein fractions were quantified using the Bradford reagent (BioRad Laboratories). Twenty micrograms of proteins was loaded onto 4% to 12% Tris-HCl gels which were run in 1 × Tris/glycine buffer, pH 8.3 (Merck) under a constant 25 mA current, and blotted onto polyvinylidene difluoride (PVDF) membranes (BioRad Laboratories) under 30 V constant voltage for 2 h at room temperature. The membranes were first incubated in blocking buffer (5% milk powder/0.1% Tween 20/PBS) for 1 h at room temperature, followed by an overnight incubation at 4°C with the following primary antibodies: anti-CD9 (1:1000; Abcam), anti-p-Akt-Ser473 (1:500), anti-p-MapK-Thr202/Tyr204 (1:2000), anti-p-Stat3-Tyr705 (1:2000) (all from Cell Signaling), and anti-GAPDH (1:2500; Abcam). A secondary IgG horse radish peroxidase antibody (1:2500; Promega) and the Amersham ECL Prime Western blotting detection reagent (GE Healthcare) were used for the chemiluminescence detection. The data were quantified using the Image J software (National Institutes of Health; http://rsbweb.nih.gov/ij/) following the developer instructions (http://rsb.info.nih.gov/ij/docs/index.html), and expressed as relative protein expression.

### shRNA silencing of *CD9* in glioblastoma stem cells

The GSCs (5 × 10^3^ cells/well) were seeded into a 48-well plate (Corning, Life Sciences) in complete Neurobasal Medium containing 8% methylcellulose (Sigma-Aldrich). Each of five different MISSION^®^ *CD9*-targeted shRNA lentiviral plasmids or a non-targeting control plasmid MISSION^®^ pLKO.1-puro Non-Mammalian shRNA with the sequence 5′-CCGGCAACAAGATGAAGAGCACC AACTCGAGTTGGTGCTCTTCATCTTGTTGTTTTT-3′ (all from Sigma–Aldrich) was added drop-wise to the cells in a well (one plasmid/well) at a volume ratio of 1:1. Stable cell lines were generated by puromycin selection (0.5 mg/L) and removal of dead cells using Dead Cell Removal Kit (Miltenyi). For further experiments, cells transduced with the following *CD9*-targeted shRNA lentiviral plasmid sequences were used: 5′-CCGGCACAAGGATGAGGTGATTAAGCTCGAGCT TAATCACCTCATCCTTGTGTTTTTG-3′ (shCD9 NCH644), 5′-CCGGCTTCGAGCAAGAAACTAATAACTCGAGTTA TTAGTTTCTTGCTCGAAGTTTTTG-3′ (shCD9 NCH421k) and 5′-CCGGCTTCGAGCAAGAAACTAATAACTCG AGTTATTAGTTTCTTGCTCGAAGTTTTTG-3′ (shCD9 NCH660h).

### Immunocytochemistry

The GSC spheroids were washed with PBS, fixed in ice-cold methanol (Sigma-Aldrich) for 15 min at room temperature, and incubated for 15 min in 0.1%Triton X-100/1% bovine serum albumin/PBS at room temperature for membrane permeabilisation. The spheroids were stained for 30 min at room temperature with the following antibodies: anti-CD133 (AC133, 1:10; Milteny), anti-nestin (1:200; EMD Millipore), anti-SOX-2 (1:100; Abgent), fibrillary acidic protein (GFAP) (1:200; Abcam), and b-III-tubulin (1:200; EMD Millipore, USA). For the negative control, the spheroids were stained with IgG1 κ isotype control protein (Abcam). Next, the spheroids were stained with an Alexa Fluor 488^®^-conjugated secondary antibody (1:200; Invitrogen, Life Technologies) for 30 min at room temperature. For nuclei staining, the spheroids were incubated with the Hoechst 33342 dye (1:1000, Invitrogen, Life Technolgies), for 5 min at room temperature. The spheroids were then mounted with AntiFade reagent (Invitrogen, Life Technologies) and analyzed under the inverted fluorescence microscope (Nikon; 200 × magnification), using the NIS-Elements Advanced Research software, version 4.13.04 (Nikon).

For the differentiation study, round glass coverslips were coated with 100 μg/mL poly-L-lysine (Sigma-Aldrich) and placed into the wells of 24-well plates (Costar, Inc.). Next, the GSCs (4 × 10^4^/well) were seeded and cultured in Neurobasal Medium containing 2 mM L-glutamine, 1x penicillin/streptomycin and 10% FBS for 10 days, with a change for fresh medium every 3 days. The attached cells were washed with PBS and prepared for immunostaining, as described above. Results of immunocytochemical labeling were quantified by computer image analysis using the ImageJ software [54]. In images obtained from the same fields of view, the Particle analysis function was applied to images obtained with UV excitation (Hoechst stain) in order to determine the total number of cells. The percentage of positive cells was determined by analyzing thresholded images obtained with blue excitation (antibody label) with the Particle analysis function. In cases when cells overlapped, positive cells were counted. Differences between groups were determined with the Chi-square test in GraphPad Prism, version 5.01 for Windows (GraphPad Software).

### Clonogenic assay

Wells of 6-well plates (Costar, Inc.) were coated with a mixture of 0.6% low-melting-point agarose (Invitrogen, Life Tech.) and complete Neurobasal Medium. The GSC spheres were dissociated into a single-cell suspension using trypsin/EDTA, and seeded into the coated wells at 2 × 10^3^ cells/well, in a mixture of 0.3% low-melting-point agarose and complete Neurobasal Medium. Additional complete Neurobasal Medium was added to the cells afterwards. The cultures were maintained under standard conditions for 21 days, with a change for fresh medium every 3 days. The formed spheroids with a diameter ≥ 100 μm were counted under the microscope (Nikon), at 100 × magnification. The number of spheroids counted was divided by the number of cells seeded, and expressed as the percentage of cells that retained the ability to form spheroids.

### Cell cycle analysis

Mechanically dissociated GSC spheroids were washed with PBS and fixed in ice-cold absolute ethanol (Sigma-Aldrich). The cells were resuspended in PI/RNase staining buffer (BD Biosciences) and incubated for 15 minutes at room temperature. Samples were measured on a FACSCalibur^TM^ (BD Biosciences), followed by analysis of data with ModFitLT Flow Cytometry Modeling Software, version 4.0 (Verity Software House).

### MTS assay

Viability of the two GSC lines, NCH644 and NCH421k, was assessed using MTS assay. GSCs were seeded in 96-well microtiter plates (Costar, Inc.) at 1 × 10^4^ cells/well in 160 μl of complete Neurobasal Medium. After a 72 hour incubation at 37°C in a 5% CO2 humidified atmosphere, the medium was carefully removed and 20 μL of MTS agent (Promega) was added to each well and incubated at 37°C for two hours. The viability of the cells was measured at 490 nm using the absorbance microplate reader Genios (Tecan). The assays were performed in duplicates and repeated in three independent experiments.

### Apoptosis assay

The GSC spheroids were washed with PBS and were dissociated into a single-cell suspension using trypsin/EDTA, followed by addition of DMEM containing 10% FBS. The cells were then pelleted by centrifugation (125 × *g*, 3min), washed in PBS, and stained with the FITC-conjugated Annexin V protein and 50 mg/L propidium iodide (both from BD Biosciences). Detection of the labeled apoptotic and necrotic cells was performed using a FACSCalibur^TM^, and analyzed with the CellQuest Pro^TM^ software, version 6.0 (BD Biosciences).

### Three-dimensional invasion assay

The GSCs were seeded in complete Neurobasal Medium containing 4% methylcellulose in 96-well plates (5 × 10^3^ cells/well; Corning, Life Sciences), centrifuged at 850 × *g* for 90 min, and then incubated at 37°C under 5% CO_2_ for 3 days, to form one spheroid in each well. These spheroids were transferred onto 24-well plates (Corning, Life Sciences) and embedded in a collagen matrix (1 mg/mL; BD Collagen Type I, rat tail). After a 45-min incubation at 37°C under 5% CO_2_, the spheroids were covered with complete Neurobasal Medium. The invasion distance was measured for 5 days under the light microscope (Nikon, 40 × magnification). The invasion distance was defined as the distance from the edge of the spheroid to the most distant cell population.

### Animal experiments

Three days prior the experiments, spheroids from 1 × 10^4^ NCH644 or NCH421k cells were prepared following the protocol for the three-dimensional invasion assay described above. Nude rats (Rowett nu/nu) were anaesthetized with Fentanyl/Dormicum (130 mg/kg/0.3 mg/kg s.c.). The rat heads were secured in a stereotactic frame (Benchmark) and a short longitudinal incision was made in the scalp, to expose the calvarium. A burr-hole was made 1 mm posterior to the bregma and 3.0 mm to the right of the sagittal suture, using a micro-drill. A Hamilton syringe with an inner diameter of 810 μm was introduced to a depth of 3.0 mm below the brain surface and 10 prepared GSC spheroids were slowly injected. The skin was closed with an Ethilon 3–0 suture. The rats were sacrificed at the onset of symptoms using CO_2_, and their brains were removed. All of the procedures involving rats in this study were approved by The National Animal Research Authority and conducted according to the European Convention for the Protection of Vertebrates Used for Scientific Purposes.

### Immunohistochemistry of rat xenografts

Paraffin-embedded tissue sections (6 μm) were prepared from the rat brains. The presence of a tumor was confirmed using Mayer's staining with hematoxylin and eosin B (both from Sigma-Aldrich), according to the standard procedures. The immunostaining on prepared sections was performed with an automated Benchmark XT platform (Ventana Medical Systems). Heat-mediated antigen retrieval was achieved with sodium citrate buffer (pH 6.0). The following anti-human primary antibodies were applied for 1 h at room temperature: anti-nestin (clone 10C2, 1:200; Millipore), anti-SOX2 (clone EPR3131, 1:50; Abgent), anti-Ki-67 (clone EPR3610, 1:200; Abcam), and anti-CD9 (clone MEM-61, 1:50; Abcam). These were followed by incubation with the secondary horse-radish-peroxidase–conjugated antibodies. Protein expression was detected using DAB substrate, and hematoxylin was used for counterstaining. The negative-control staining was performed without the addition of the primary antibodies. Staining evaluation was carried out under an upright light microscope (Nikon) by two independent investigators. The evaluation of data was not carried out as double blind. The complete expression scores were calculated as described by Vrzalikova et al. [55] but without giving scores to the percentages of positive cells. Briefly, mean percentage of positive cells in 10 visual fields throughout the whole tumor area was multiplied by the mean of the staining intensity, as: 0, negative; 1, weak; 2, moderate; 3, strong.

### Statistical analysis

One-way and two-way ANOVA tests with post-Bonferroni tests were used to detect significant differences between the test groups. All of the statistical analyses were performed using GraphPad Prism, version 5.01 for Windows (GraphPad Software). P value < 0.05 was considered significant. All of the *in-vitro* experiments were repeated independently three times.

## SUPPLEMENTARY FIGURES AND TABLES










