# Supplementary material for: Transmembrane protein CD9 is glioblastoma biomarker, relevant for maintenance of glioblastoma stem cells
Source: Oncotarget. 2015 Nov 11;7(1):593–609. doi: 10.18632/oncotarget.5477 (PMC4808020; doi:10.18632/oncotarget.5477)
Supplement: Supplementary file 1 [file oncotarget-07-0593-s001.pdf]

SUPPLEMENTARY FIGURES AND TABLES

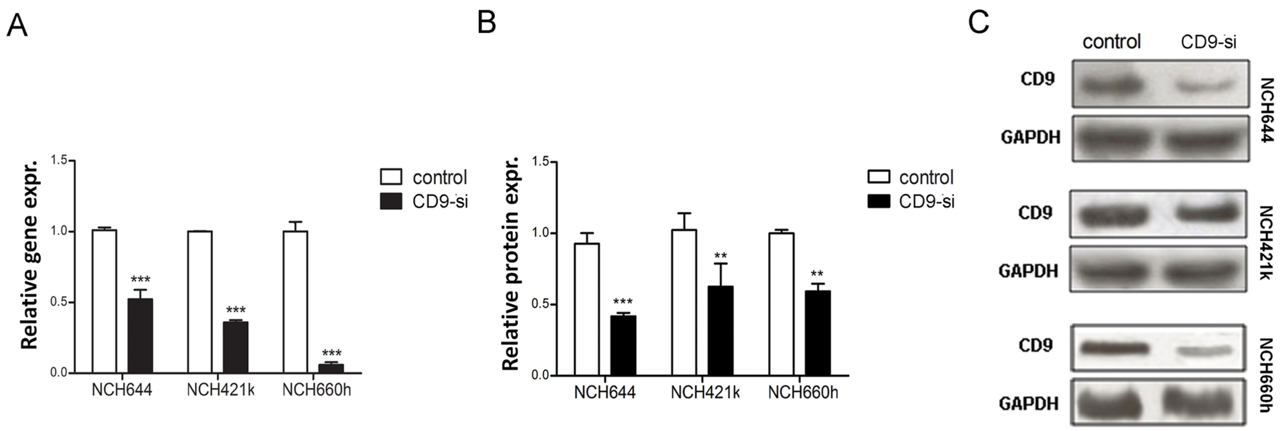

Supplementary Figure S1: *CD9* silencing decreased its expression at gene and protein levels. Three GSC lines (NCH644, NCH421k and NCH660h) were targeted for *CD9* expression using lentiviral shRNA against *CD9* ("CD9-si"). For controls, the cells were transduced with a non-targeting vector ("control"). **A.** Analysis by qPCR for relative *CD9* expression in three GSC lines upon *CD9* silencing. ***, $p < 0.001$. **B.** Quantification of Western blotting for relative CD9 expression in three GSC lines upon *CD9* silencing. **, $p < 0.01$; ***, $p < 0.001$. **C.** Representative Western blotting of CD9 expression in GSC lines upon *CD9* silencing. GAPDH is included as an internal control.

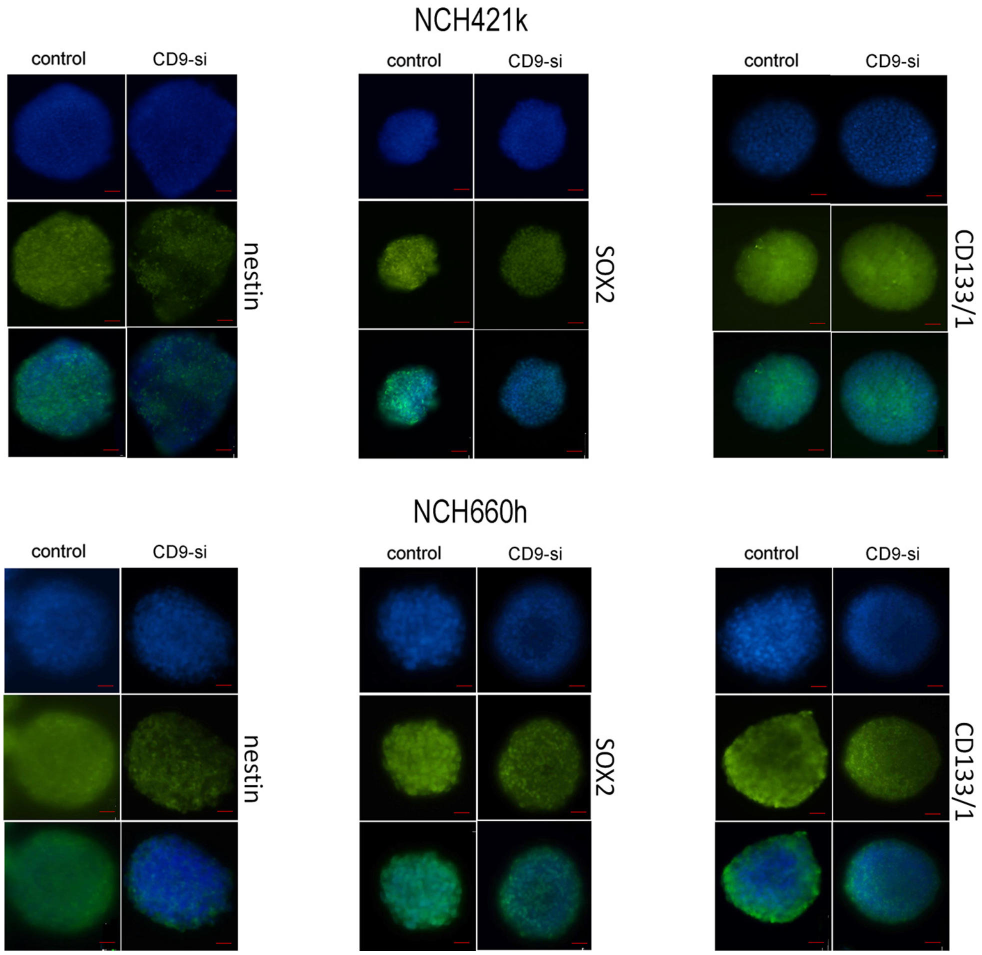

Supplementary Figure S2: *CD9* silencing reduced stem cell marker expression. Nestin, SOX2 and Cd133/prominin-1 protein expression analysis in the *CD9*-silenced NCH421k and NCH660h cell lines. Blue, cell nuclei; green, proteins, as indicated. Representative images of three repeated experiments are shown for both channels (blue, green) as well as for overlays. Scale bar, 100 µm.

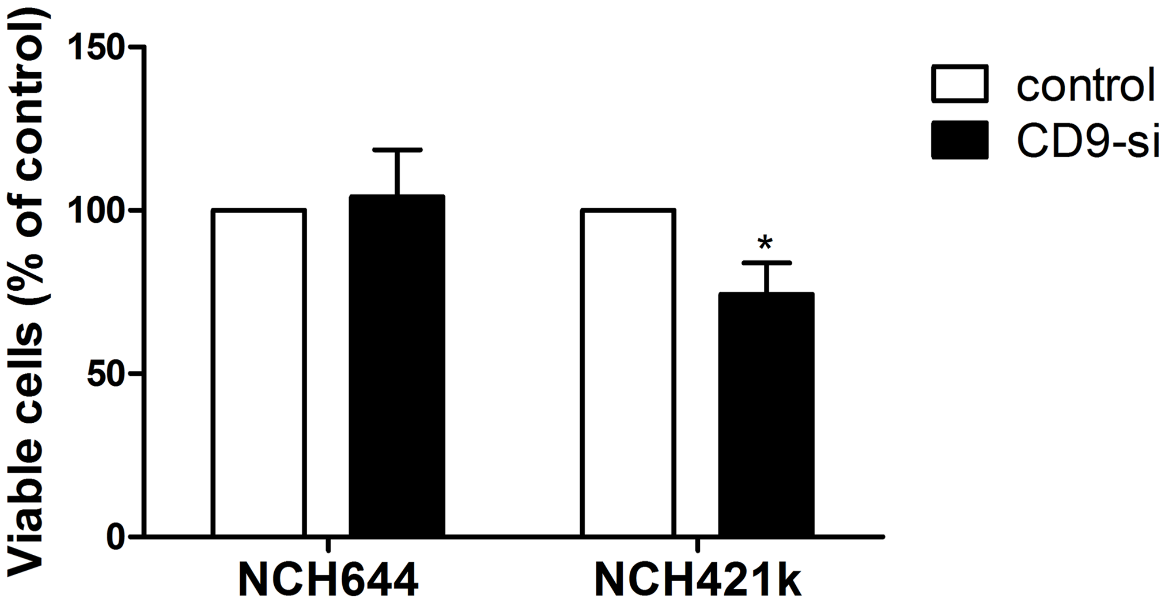

Supplementary Figure S3: *CD9* silencing decreased viability of GSCs. The effects of *CD9* silencing on viability of the GSC lines ("CD9-si") was measured by MTS assay and compared to the non *CD9*-silenced cells ("control"). Data are means ±SD of three independent experiments. *, $p < 0.05$.

Supplementary Table S1: *CD9* is not up-regulated in the U87-MG cell line when compared to the normal human astrocytes. Transcriptomic analyses were performed with the data obtained from the publicly available GEO and EMBL-EBI ArrayExpress for U87-MG versus NHAs. CD9 was not found among the 591 de-regulated genes in the U87-MG versus the NHAs.

Supplementary Table S2: *CD9* is up-regulated in the U373 cell line when compared to the normal human astrocytes. Transcriptomic analyses were performed with the data obtained from the publicly available GEO and EMBL-EBI ArrayExpress for U373 versus NHAs. CD9 was found to be up-regulated among the 564 de-regulated genes in U373 versus the NHAs.

Supplementary Table S3: *CD9* is up-regulated in GBM tissue when compared to the normal brain. Transcriptomic analyses were performed with the data obtained from the publicly available database of TCGA, for GBM tissue versus normal brain tissue. CD9 was found to be up-regulated among the 3521 de-regulated genes in GBM tissue versus normal brain tissue.

Supplementary Table S4: Overview of selected genes up-regulated in GBM cell lines and tissues when compared to their normal counterparts. All de-regulated genes were ranked in groups based on their expression in GBM cells and / or tissue, cellular localisation and their connection with EGFR and / or FGFR.
